# Case Report: New CDKN1B Mutation in Multiple Endocrine Neoplasia Type 4 and Brief Literature Review on Clinical Management

**DOI:** 10.3389/fendo.2022.773143

**Published:** 2022-03-09

**Authors:** Elisabetta Lavezzi, Alessandro Brunetti, Valeria Smiroldo, Gennaro Nappo, Vittorio Pedicini, Eleonora Vitali, Giampaolo Trivellin, Gherardo Mazziotti, Andrea Lania

**Affiliations:** ^1^ Endocrinology, Diabetology and Andrology Unit, IRCCS Humanitas Research Hospital, Rozzano, Italy; ^2^ Department of Biomedical Sciences, Humanitas University, Pieve Emanuele, Italy; ^3^ Oncology Unit, IRCCS Humanitas Research Hospital, Rozzano, Italy; ^4^ Pancreatic Surgery Unit, IRCCS Humanitas Research Hospital, Rozzano, Italy; ^5^ Radiology Unit, IRCCS Humanitas Research Hospital, Rozzano, Italy; ^6^ Endocrinology Unit and Laboratory of Cellular and Molecular Endocrinology, IRCCS Humanitas Research Hospital, Rozzano, Italy

**Keywords:** men, multiple endocrine neoplasia, CDKN1B, familiar, hyperparathyroidism, neuroendocrine tumor, pituitary adenoma

## Abstract

**Background:**

The fourth type of multiple endocrine neoplasia (MEN) is known as a rare variant of MEN presenting a MEN1-like phenotype and originating from a germline mutation in CDKN1B. However, due to the small number of cases documented in the literature, the peculiar clinical features of MEN4 are still largely unknown, and clear indications about the clinical management of these patients are currently lacking. In order to widen our knowledge on MEN4 and to better typify the clinical features of this syndrome, we present two more cases of subjects with MEN4, and through a review of the current literature, we provide some possible indications on these patients’ management.

**Case Presentation:**

The first report is about a man who was diagnosed with a metastatic ileal G2-NET at the age of 34. Genetic analysis revealed the mutation p.I119T (c.356T>C) of exon 1 of CDKN1B, a mutation already reported in the literature in association with early-onset pituitary adenomas. The second report is about a 76-year-old woman with a multifocal pancreatic G1-NET. Genetic analysis identified the CDKN1B mutation c.482C>G (p.S161C), described here for the first time in association with MEN4 and currently classified as a variant of uncertain significance. Both patients underwent biochemical and imaging screening for MEN1-related diseases without any pathological findings.

**Conclusions:**

According to the cases reported in the literature, hyperparathyroidism is the most common clinical feature of MEN4, followed by pituitary adenoma and neuroendocrine tumors. However, MEN4 appears to be a variant of MEN with milder clinical features and later onset. Therefore, these patients might need a different and personalized approach in clinical management and a peculiar screening and follow-up strategy.

## Introduction

Multiple endocrine neoplasia (MEN) is a rare genetic syndrome that predisposes patients to develop tumors in one or more endocrine organs. Depending on the endocrine glands most frequently involved, MEN is classified into different categories, numbered from 1 to 4. MEN1 is mainly characterized by primary hyperparathyroidism, pituitary adenomas, and neuroendocrine tumors (NETs) and is caused by a mutation in menin. MEN2A is characterized by medullary thyroid carcinoma, pheochromocytoma, and hyperparathyroidism, and MEN2B by medullary thyroid carcinoma, pheochromocytoma, ganglioneuromas, and musculoskeletal anomalies. Both MEN2A and MEN2B originate from a mutation in the RET oncogene. The fourth type of multiple endocrine neoplasia is the most recently introduced (1) and includes subjects phenotypically similar to MEN1 but not carrying any germline mutation in the the menin gene. This condition, previously known as MENX, was first reported in humans by Pellegata et al. (2), who described it as a potential cause of mutation of the CDKN1B gene.

CDKN1B encodes for the p27 protein, an important regulator of the cell cycle, which plays a pivotal role in a wide range of cellular activities such as inhibition of cyclin-dependent kinase, regulation of apoptosis, and interaction with the cytoskeleton (3). Heterozygous mutations in CDKN1B have been proven to encode for a p27 protein with either a truncated structure or reduced binding activity, leading to loss of major tumor-suppressor functions (4). As such, CKDN1B is thought to act as a tumor suppressor through a haploinsufficiency mechanism.

CDKN1B mutations have been associated with a wide variety of endocrine and non-endocrine neoplasms such as luminal breast cancer (5), prostate cancer (6), and hairy cell leukemia (7). However, the specific mechanism promoting the development of a MEN1-like phenotype is still unclear. Menin physiologically regulates both CDKN1B transcription and p27 expression through epigenetic factors, and its inactivation was found to induce a p27 downregulation (8). Therefore, tumor development in MEN1 and MEN4 could share a common pathway (9).

To date, 23 different mutations of CDKN1B have been described in the literature in association with MEN4, including a total of 57 carriers. Forty-two of these subjects developed at least one endocrine neoplasm, while the involvement of multiple endocrine organs was detected in 17 of them (**Table 1**). Phenotypic and haplotypic analyses of the carriers’ families suggest that MEN4 follows an autosomal dominant pattern of transmission, although disease penetrance is not yet completely known. Primary hyperparathyroidism is reported as the most common clinical feature, followed by pituitary adenomas and neuroendocrine tumors. In addition, several non-endocrine neoplasms have also been described, such as breast cancer, prostate cancer, colon cancer, papillary thyroid carcinoma, angiomyolipoma, meningioma, and adrenal adenoma.

**Table 1:** Studies describing families with MEN4 syndrome associated with CDKN1B mutations.

Mutation	Type of mutation	Carriers with MEN4-associated features			Asymptomatic family carriers (age at assessment)	Total carriers	Reference
		Sex	Main MEN features (age at diagnosis)	Other significant history of neoplasia			
c.G692A (p.W76X)	Nonsense	F	GH-producing pituitary macroadenoma (30 years old) Primary hyperparathyroidism (PHPT) (46 years old)	NO	One sister without MEN main features but with a history of angiomyolipoma diagnosed at 52 years old. Another sister (44 years old) and her daughter (16 years old) completely asymptomatic.	4	Pellegata et al. (2)
c.59_77dup19 (pK25fs)	Frameshift	F	Small-cell neuroendocrine cervical carcinoma (45 years old) ACTH-secreting pituitary adenoma (46 years old) PHPT (47 years old)	NO	None	1	Georgitisi et al. (10)
ATG-7G>C	Mutation in 5′UTR	F	PHPT (61 years old)	Bilateral NF adrenal masses (63 years old)	2 daughters (46 and 48 years old)	3	Agarwal et al. (11)
c283C>T (p.P95S)	Missense	F	PHPT with multiple adenoma (46 years old), metastatic gastrinoma (50 years old)	NO	None	1	Agarwal et al. (11)
c.595T>C (p.*199Qext60)	Nonsense	F	PHPT with multiple adenoma (50 years old)	NO	None	2	Agarwal et al. (11)
		F	PHPT (66 years old)	NO			Agarwal et al. (11)
c.163G>A (p.Ala55Thr)	Missense	F	Metastatic gastrinoma (42 years old) PHPT (51 years old)	NO	None	1	Belar et al. (12)
c.678C>T (p.P69L)	Missense	F	PHPT (67 years old) Metastatic bronchial carcinoid (67 years old) Non-functioning pituitary microadenoma (79 years old)	Papillary thyroid carcinoma (64 years old)	None	1	Molatore et al. (13)
c.-456_-453delCCTT	Deletion in 5′UTR	F	GH-secreting adenoma (62 years old) Non-functioning pancreatic NET (62 years old)	NO	None	1	Occhi et al. (14)
c.374_375delCT	Frameshift	F	PHPT from multiple adenoma (41 years old) Metastatic gastrinoma (50 years old)	NO	1 son (34 years old)	2	Tonelli et al. (15)
c.25G>A (Gly9Arg)	Missense	M	PHPT (68 years old)	NO	None	1	Costa-Guda et al. (16)
c.397CA (p.Pro133Thr)	Missense	F	PHPT (53 years old)	NO	None	3	Costa-Guda et al. (16)
		F	PHPT (56 years old)	Papillary thyroid carcinoma (56 years old) Uterine leiomyoma (NA) Grade II meningioma (NA)	None		Bugalho and Domingues (17)
		F	PHPT from multiple adenoma(49 years old) Mutation also in MEN1	Fibrocystic breast disease (NA)	None		Borsari et al. (8)
c.-80C>T	Missense	M	PHPT (38 years old)	NO	None	1	Borsari et al. (8)
c.-29_-26delAGAG	Deletion in 5′UTR	F	PHPT (61 years old)	NO	None	5	Borsari et al. (8)
		F	GH-secreting pituitary adenoma (5 years old)	NO	Mother (age not reported)		Sambugaro et al. (18)
		F	Gastric carcinoid tumor (69 years old) PHPT (74 years old)	NO	None		Malanga et al. (19)
		M	ACTH-secreting pituitary microadenoma (13 years old)	NO	None		Chasseloup et al. (20)
c378G>C (pE126D)	Missense	F	PHPT (15 years old)	NO	Mother (46 years old) Grandfather (74 years old)	3	Elston et al. (21)
c.320delA (p.Q107Rfs*12)	Frameshift	F	ACTH-secreting pituitary microadenoma (16 years old)	NO	4 (mother, brother, and 2 children)	5	Chasseloup et al. (20)
c.376G>C (p.E126Q)	Missense	F	ACTH-secreting pituitary microadenoma (12 years old)	NO	None	1	Chasseloup et al. (20)
c.407A>G (p.D136G)	Missense	M	ACTH-secreting pituitary microadenoma (15 years old)	NO	None	1	Chasseloup et al. (20)
c.356T>C (p.I119T)	Missense	F	ACTH-secreting pituitary microadenoma (15 years old)	NO	None	2	Chasseloup et al. (20)
		F	GH-secreting adenoma (NA)	NO	(aunt with GH-secreting adenoma, not tested for CDKN1B mutation)		Tichomirowa et al. (22)
c.286AOC (p.K96Q)	Missense	F	PRL-secreting adenoma (NA)	Breast cancer (41 years old)	1 sister (age not reported)	2	Tichomirowa et al. (22)
c.285dupC (Lys96Glnfs*29)	Duplication	M	PHPT (60 years old)	Positive for NF1. Prostate cancer with transdifferentiation in metastatic small cell carcinoma (60 years old) Multiple spinal neurofibromas	None	1	Brock et al. (23)
c.281C>T, (Pro94Leu)	Missense	F	Hyperprolactinemia (adenoma non-detected at MRI) (35 years old) PHPT (51 years old)	Breast cancer (37 years old) Ovarian serous cystadenoma (50 years old) Multifocal papillary thyroid carcinoma (55 years old)	None	1	Chevalier et al. (24)
c.206C>T, p. (Pro69Leu9)	Missense	F	Non-functioning macroadenoma (66 years old) PHPT (66 years old)	Subclinical cortisol autonomous secretion by adrenal adenoma (NA)	None	1	Chevalier et al. (24)
c.121_122 delTT (pLeu41Asnfs*83	Frameshift	F	PHPT and ACTH-secreting pituitary microadenoma (37 years old)	NO	All the patients belong to the same family, no other healthy carrier reported	13	Frederkisen et al. (25)
		F	PHPT and non-functioning pancreatic NET (67 years old)	NO			
		F	PHPT and non-functioning pituitary microadenoma (66 years old)	NO			
		M	PHPT and non-functioning pituitary microadenoma (64 years old)	NO			
		M	PHPT and non-functioning pituitary macroadenoma (46 years old)	NO			
		F	PHPT- and ACTH-secreting pituitary microadenoma (NA)	NO			
		5F + 2M	PHPT (NA)	One F developed had a recurrency of PHPT after first surgery and had breast cancer (77 years old)			

M, male; F, female; PHPT, primary hyperparathyroidism; NA, not assessed.

Due to the small number of cases reported so far, it is still unclear whether MEN4 should be considered only as a rare variant of MEN1 or whether it presents some distinctive clinical features. Furthermore, clear guidelines on the clinical management of these patients are lacking. In a recent review, Frederiksen et al. have suggested screening these patients for hyperparathyroidism and pituitary tumors in adolescence and performing screening for NETs according to the guidelines provided for MEN1 (25).

In this report, we describe two patients developing a neuroendocrine tumor and carrying a germline mutation in CDKN1B. Moreover, through a new review of the most recent cases in the literature, we try to start outlining the first differences between MEN1 and MEN4 and to provide new indications on the clinical management of these patients.

## Case Description

### Case 1

#### Clinical History

The first case is a 43-year-old man with no significant previous medical history, who was diagnosed with a metastatic ileal G2-NET at the age of 34. The tumor presented at diagnosis as an ≈3-cm ileal mass with multiple metastasis at the lymph nodes, mesentery, and liver. Histological examination of the ileal mass, removed by segmental ileal resection as conditioning a strict stenosis, documented the following: infiltration of the entire wall from the mucous membrane to the subserous adipose tissue; mitosis number: 1 × 10 HPF; Ki67: about 3%; and presence of diffuse neoplastic permeation of lymphatic vessels, both peritumoral and distant. Due to the widespread localization of the disease, treatment with first-generation somatostatin receptor ligands (SRLs) was started. After almost 10 years of substantial stability of disease in SRL therapy, recent 68-Ga-DOTATOC PET and abdomen MRI scans documented a progression. As such, the patient is currently being evaluated for peptide receptor radionuclide therapy.

#### Genetic and Clinical Testing

In consideration of the multiple localizations of the disease at a relatively young age, the patient was also advised to undergo a genetic evaluation for MEN. The analysis was performed through next-generation sequencing (NGS) (Illumina, Inc.Hayward, CA, USA: Illumina-Nextera Rapid Capture Custom Enrichment kit) of coding regions and intron/exon junctions of AIP, CDC73, CDKN1B, MEN1, and PRKAR1a.

The analysis identified the mutation p.I119T (c.356T>C) in heterozygosity of exon 1 of CDKN1B. This mutation was identified in control databases in 152 of 279,092 alleles at a frequency of 0.0005446 (gnomAD). *In-silico* analysis does not predict a difference in splicing. This variant was determined to be of uncertain significance according to ACMG guidelines, 2015, and had already been reported in the literature in association with early-onset pituitary adenoma (20, 22).

The patient was then offered screening for MEN1-related diseases, including assessment of parathyroid function (blood samples for PTH, calcium, phosphorus, albumin, 25OH vitamin D) and pituitary function (blood samples for TSH, FT4, IGF-1, ACTH, prolactin, FSH, LH, testosterone, morning cortisol at h 8 a.m. and after stimulation with 1 mcg ACTH test), and MRI of sella turcica. All the examinations reported no significant findings.

#### Family History

Genetic evaluation was then extended to the patient’s relatives, with positive findings in the father and younger brother. The same screening for MEN1 was then offered to all family carriers, with the addition of an abdominal ultrasound (not performed in the index patient because of periodic CT follow-up).

The patient’s brother, aged 31, had no significant medical history and no symptoms. Sellar MRI and abdominal ultrasound documented no pathological findings. Parathyroid and pituitary function resulted normal as well.

The patient’s father, aged 70, had a history of clear-cell renal carcinoma diagnosed at the age of 57, in complete remission after unilateral nephrectomy and adrenalectomy. Screening for MEN1-related diseases revealed also, in this case, no significant findings at MRI, abdomen ultrasound, and pituitary and parathyroid function assessments.

### Case 2

#### Clinical History

We also present the case of a 76-year-old woman, reporting in medical history only an ovarian dermoid cyst removed when she was 39. At the age of 73, during a routine abdominal ultrasound, an ≈3-cm pancreatic mass was detected. A subsequent CT scan confirmed the presence of a 3.2-cm solid and partially hypervascular mass in the pancreatic uncinate process, suggestive of NET. The diagnosis of NET was supported by enhanced uptake at 68-Ga-DOTATOC PET and confirmed through endoscopic ultrasound biopsy. No further localization of the disease was observed, so the patient underwent the Whipple procedure with complete remission of the disease. At the last 3-year follow-up, there was no evidence of disease recurrence.

Histological examination of the mass documented “three different foci of G1-NET of the pancreatic head (according to WHO 2010), respectively, 2.9, 0.6, and 0.5 cm; Ki67: 1%–2%; mitotic index: <1 mitosis/10 HPF; angioinvasion present, no necrosis or neural invasion detected; and no lymph nodes involved”.

#### Genetic and Clinical Testing

Due to the multifocality of the disease, the patient was advised for genetic testing, performed as described in case 1. The analysis showed a missense heterozygous variant in exon 2 of CDKN1B (c.482C>G, p.S161C). This mutation has never been reported so far in any patient with MEN4 phenotype. It is present in population databases (rs373917399, ExAC 0.04%) and has an allele count higher than expected for a pathogenic variant (PMID: 28166811). The Genome Aggregation Database (gnomAD) reported an allele frequency of 0.00014. The variant is located into the C-terminal RhoA binding domain (26). Since p27 modulates actin dynamics by direct regulation of the RhoA pathway (27), this variant could affect the interaction between p27 and RhoA and the consequent regulation of actin dynamics and cell motility. However, *in-silico* analysis supports that this missense variant does not alter protein structure/function, and thus, it has been currently classified as a variant of uncertain significance.

The patient was then offered screening for MEN-related diseases, as described in case 1. Sellar MRI documented partial empty sella, but no pituitary function impairment was detected at biochemical exams. Parathyroid function resulted normal as well.

#### Family History

Genetic evaluation was extended to the patient’s family, with a positive finding in the patient’s 76-year-old sister. She was also offered a screening test for MEN-related diseases, but she refused to undergo any examination because she was not reporting any symptoms.

## Discussion

MEN4, although basically recognized as a variant of MEN1, is still a condition whose clinical features are relatively unknown, mainly because of the small number of cases in the literature. However, with the increase of MEN4 reports, we have the opportunity to better characterize its similarities with MEN1, but also to begin to delineate the differences.

Both MEN1 and MEN4, despite an autosomal dominant transmission which theoretically predicts an equal distribution in both sexes, are more common in women (57% MEN1, 75% MEN4). The significantdifference in gender prevalence reported in MEN4 could still be due to the small population examined. However, it confirms a trend also present in MEN1 of greater penetrance in the female sex, whose causes are still not clear (28).

Primary hyperparathyroidism results in MEN4, as in MEN1, the most frequent endocrine neoplasm. However, in MEN4, the prevalence seems to be lower (75% vs. >93%) and with a more advanced mean age at diagnosis (53 vs. 40 years) (28). According to case reports in the literature, the youngest age of onset is 15 years in MEN4, while in MEN1, primary hyperparathyroidism might occur at a much earlier age (as young as 5 years) (29). Thus, as suggested by Frederiksen et al. (25), screening for hyperparathyroidism in subjects with MEN4 might start in adolescence and not in childhood as MEN1. Moreover, in MEN4, hyperparathyroidism is more frequently caused by one single parathyroid adenoma, while the involvement of multiple parathyroids and/or recurrence after surgery is quite rare (only five cases documented). Therefore, in patients with MEN4, we suggest that the surgical approach could be limited to the removal of the single hyperfunctioning parathyroid without the necessity of subtotal parathyroidectomy.

The prevalence of pituitary adenoma in MEN4 is around 40%, substantially overlapping with MEN1 (28). Pituitary adenomas may affect subjects of all ages; although the mean age is 33–35 years, the youngest case report is a 5-year-old girl. Therefore, we suggest assessing basal pituitary function in any MEN4 patient and eventually performing an MRI of sella turcica if the hormonal profile should prove abnormal. Interestingly, although prolactinomas are the most common form of functioning adenoma in MEN1, in MEN4, they seem to be the rarest type, with only one case reported. In contrast, corticotropinomas, which represent only 5% of the cases in MEN1 (28), appear to account for almost 40% of all pituitary adenomas in MEN4.

Currently, it is not possible to determine whether this difference is due to a statistical issue related to the small number of cases or to a specific effect of CDKN1B on pituitary tumor development.

Neuroendocrine tumors occur in ≈20% of MEN4 subjects, a significantly lower percentage than MEN1 (≈50%). The mean age is around 55 years, and the youngest so far is our patient at the age of 34. Localization of the primary tumor includes the pancreas, small intestine, and lung. A small cell neuroendocrine tumor of the cervix was also described in one woman (10). The most common form of functioning NET is gastrinoma, as in MEN1, while no cases of VIPomas, glucagonomas, insulinomas, or somatostatinomas have been reported so far.

The significant difference in NET prevalence between MEN1 and MEN4 could be due to discrepancies in the clinical assessment of patients. In MEN1, it is indeed recommended to investigate the presence of neuroendocrine tumors even in asymptomatic subjects, performing periodic biochemical and imaging investigations (30). However, there are no such clear guidelines for MEN4 patients, so the cases reported so far may not have been screened for NETs as accurately as in MEN1, especially if the patient did not show any symptoms.

In agreement with Frederiksen et al., considering the severe comorbidity associated with NETs and the lack of conclusive data on the real prevalence in MEN4, we suggest that all MEN4 patients should be screened for NETs according to the same guidelines as for MEN1.

Moreover, in MEN4, also non-endocrine neoplasms have been reported such as breast cancer, prostate cancer, colon cancer, angiomyolipoma, meningioma, and adrenal adenoma. Many of these conditions have also been reported in MEN1 (28). In addition, CDKN1B has been recognized as a potential driver mutation in the development of breast cancer and prostate cancer (31). Recent studies have also highlighted the predisposition of Cdkn1b-mutated rats in developing pheochromocytoma (32), although no cases have been reported in humans yet.

Finally, we must consider that, despite the wide variety of neoplasms associated with CDKN1B, the true oncogenic risk in CDKN1B-mutated carriers is still unknown. Most of the patients developed isolated neoplasms, difficult to distinguish from sporadic forms, and only rarely manifested a true MEN. Moreover, a significant number of CDKN1B mutation carriers were totally asymptomatic. As such, we cannot exclude that CDKN1B mutation alone may not be sufficient to determine a clinically overt MEN, but other factors might be involved in triggering tumorigenesis in these patients.

## Conclusions

Thanks to the increased number of cases reported on MEN4, it is possible to more accurately define both similarities and differences in comparison with MEN1. MEN4 appears to be a variant with a later onset, less penetrance, and milder clinical features. Hyperparathyroidism is the most common clinical feature, although in our cases this condition was not found; since recurrence and/or multiple parathyroid involvement appears to be rare, a less aggressive surgical approach than in MEN1 could be justified. We also suggest that all MEN4 carriers, even asymptomatic, should be screened for neuroendocrine tumors, considering that this could also represent the only pathological manifestation, as we report in our cases. Even if not reported in our patients, according to the literature, MEN4 patients should also be screened for pituitary adenomas. In conclusion, larger case series are needed to clarify the peculiar features of MEN4, to establish a specific diagnostic and therapeutic standard, and to set up an appropriate follow-up strategy. Moreover, specific studies performed on CDKN1B carriers are needed to assess the real oncological risk in these subjects and to elaborate a standardized screening protocol.

## Data Availability Statement

The raw data supporting the conclusions of this article will be made available by the authors, without undue reservation.

## Ethics Statement

The studies involving human participants were reviewed and approved by the Ethical Board IRCCS Humanitas Research Hospital. The patients/participants provided their written informed consent to participate in this study. Written informed consent was obtained from the individual(s) for the publication of any potentially identifiable images or data included in this article.

## Author Contributions

EL and AB conceived and designed the study. Data collection, analysis, and interpretation were done by AB. All authors contributed to the article and approved the submitted version.

## Conflict of Interest

The authors declare that the research was conducted in the absence of any commercial or financial relationships that could be construed as a potential conflict of interest.

## Publisher’s Note

All claims expressed in this article are solely those of the authors and do not necessarily represent those of their affiliated organizations, or those of the publisher, the editors and the reviewers. Any product that may be evaluated in this article, or claim that may be made by its manufacturer, is not guaranteed or endorsed by the publisher.
